# Chronic Ethanol Exposure Enhances Facial Stimulation-Evoked Mossy Fiber–Granule Cell Synaptic Transmission *via* GluN2A Receptors in the Mouse Cerebellar Cortex

**DOI:** 10.3389/fnsys.2021.657884

**Published:** 2021-08-02

**Authors:** Bing-Xue Li, Guang-Hui Dong, Hao-Long Li, Jia-Song Zhang, Yan-Hua Bing, Chun-Ping Chu, Song-Biao Cui, De-Lai Qiu

**Affiliations:** ^1^Brain Science Research Center, Yanbian University, Yanji, China; ^2^Department of Physiology and Pathophysiology, College of Medicine, Yanbian University, Yanji, China; ^3^Department of Neurology, Affiliated Hospital of Yanbian University, Yanji, China

**Keywords:** cerebellar cortex, sensory stimulation, mossy fiber-granule cell synaptic transmission, *in vivo* electrophysiological recording, N-methyl-D-aspartate receptors, chronic ethanol exposure

## Abstract

Sensory information is transferred to the cerebellar cortex *via* the mossy fiber–granule cell (MF–GC) pathway, which participates in motor coordination and motor learning. We previously reported that chronic ethanol exposure from adolescence facilitated the sensory-evoked molecular layer interneuron–Purkinje cell synaptic transmission in adult mice *in vivo*. Herein, we investigated the effect of chronic ethanol exposure from adolescence on facial stimulation-evoked MF–GC synaptic transmission in the adult mouse cerebellar cortex using electrophysiological recording techniques and pharmacological methods. Chronic ethanol exposure from adolescence induced an enhancement of facial stimulation-evoked MF–GC synaptic transmission in the cerebellar cortex of adult mice. The application of an N-methyl-D-aspartate receptor (NMDAR) antagonist, D-APV (250 μM), induced stronger depression of facial stimulation-evoked MF–GC synaptic transmission in chronic ethanol-exposed mice compared with that in control mice. Chronic ethanol exposure-induced facilitation of facial stimulation evoked by MF–GC synaptic transmission was abolished by a selective GluN2A antagonist, PEAQX (10 μM), but was unaffected by the application of a selective GluN2B antagonist, TCN-237 (10 μM), or a type 1 metabotropic glutamate receptor blocker, JNJ16259685 (10 μM). These results indicate that chronic ethanol exposure from adolescence enhances facial stimulation-evoked MF–GC synaptic transmission *via* GluN2A, which suggests that chronic ethanol exposure from adolescence impairs the high-fidelity transmission capability of sensory information in the cerebellar cortex by enhancing the NMDAR-mediated components of MF–GC synaptic transmission in adult mice *in vivo*.

## Introduction

Ethanol is the most widely used and abused psychoactive substance and can cause damage to the central nervous system that leads to impairment of its function. The cerebellum is a major target of ethanol. Ethanol exposure causes alterations in behavior, motor coordination, speech, balance, and cognitive functions, which are considered to be induced by the impaired function of cerebellar neuronal circuits and synaptic transmission (Schmahmann and Sherman, 1997; Mameli et al., 2008; Luo, 2012).

*In vitro*, acute ethanol exposure was shown to increase the frequency of miniature and spontaneous inhibitory postsynaptic currents in cerebellar Purkinje cells (PCs) and molecular layer interneurons by enhancing γ-aminobutyric acid (GABA) release in rats (Mameli et al., 2008; Wadleigh and Valenzuela, 2012). *In vivo*, cerebellar surface application of ethanol was shown to modulate the facial stimulation evoked by γ-aminobutyric acid-ergic (GABAergic) responses in the mouse cerebellar molecular layer (Cui et al., 2014). Acute overdose ethanol exposure inhibited the facial stimulation-evoked outward current by activating cannabinoid receptor 1 *via* the protein kinase A signaling pathway in the mouse cerebellar cortex (Wu et al., 2016). In contrast, chronic ethanol exposure impairs neuronal function, reduces the number of neurons *via* activation of N-methyl-D-aspartate receptors (NMDARs) (Nagy, 2004), and significantly inhibits the simple spike and complex spike activities of PCs (Servais et al., 2005). Chronic intermittent ethanol exposure significantly reduces the abundance of myelin sheath proteins and enzymes in the cerebellum, the corpus callosum, and the spinal cord (Samantaray et al., 2015). Chronic ethanol exposure also impairs sensory stimulation-induced molecular layer interneuron–PC long-term depression *via* the activation of the nitric oxide signaling pathway (Li et al., 2019) and significantly facilitates sensory stimulation-evoked molecular layer interneuron–PC synaptic transmission *via* the nitric oxide signaling pathway in the mouse cerebellar cortex (Sun et al., 2020).

Cerebellar granule cells (GCs) are the main integrators and processors of input information in the cerebellar cortex (Hamann et al., 2002; Duguid et al., 2012) and are considered to decorrelate the information conveyed *via* convergent multimodal mossy fibers (MFs), increasing the use for learned associations (Billings et al., 2014; Cayco-Gajic et al., 2017). Sensory information is derived from MFs, which induce excitatory responses in cerebellar GCs and are involved in the modulation of the command output of PCs (Eccles et al., 1967; Jakab and Hámori, 1988). Passive movement of the forelimb without touching the receptive field can evoke spike firing in the GCs, which indicates that the sensory information is encoded by spike firing of GCs (Jörntell and Ekerot, 2006). GCs are relatively simple spike encoders that exhibit a relatively linear conversion of the depolarization level to spike firing frequency (D’Angelo et al., 1998; Jörntell and Ekerot, 2006). Cerebellar GCs comprise a low-noise, sparse coding system that can reliably relay sensory-evoked MF signals and filter out information not associated with sensory stimulation (Chadderton et al., 2004). The spike output of GCs could reflect the sensory information coding principal during the low-intensity rate-coded MF activation (Arenz et al., 2008). GCs exhibit high-frequency and high-fidelity properties in response to sensory stimulation, which ensures the transmission of accurate sensory information to PCs (van Beugen et al., 2013; Bing et al., 2015b). In addition, GCs activate Golgi cells *via* parallel fibers (Cesana et al., 2013), which inhibit GCs *via* Golgi axon branches (Mapelli et al., 2009). In the cerebellar granular layer (GL), ethanol mediates phase and tense GABAergic inhibition in GCs by activating Golgi cells (Botta et al., 2007) and inhibits sensory stimulation-evoked responses in cerebellar GCs by enhancing GABA_A_ receptor activity (Wu et al., 2014). Chronic ethanol exposure causes a significant reduction in the number of GCs or in the total volume of the GL in adult rats (Tabbaa et al., 1999; Pentney et al., 2002). Adolescents are more sensitive to ethanol exposure than adults (Spear, 2016), especially in the cerebellum (Best and Miller, 2010). During the adolescent developmental period, chronic ethanol consumption leads to many adverse effects, such as impaired learning, attention and behavior (Maldonado-Devincci et al., 2021). Chronic ethanol exposure in adolescents has been shown to damage the cognitive flexibility of rats in adulthood (Semenova, 2012) and to impair the expression of long-term synaptic potentiation (Goodwani et al., 2017).

Cerebellar GCs exhibit high-frequency and high-fidelity properties during sensory information encoding and transfer to PCs, which are critical to motor regulation and motor learning behavior because of the modulation of the output of PCs. Chronic ethanol exposure from the adolescent stage could damage the cerebellar function by impairing the synaptic transmission of sensory information from MFs to GCs. Therefore, in this study, we studied the effects of chronic ethanol exposure in adolescence on facial stimulation-evoked MF–GC synaptic transmission in the cerebellar cortex of mice.

## Materials and Methods

### Animals

A total of 96 (5-week-old) ICR mice were selected, and they were divided into the chronic ethanol exposure group (48 mice; 22 female, 26 male) and the control group (48 mice; 23 female, 25 male). The experimental procedures were approved by the Animal Care and Use Committee of the Yanbian University and were in accordance with the animal welfare guidelines of the United States National Institutes of Health. The permit number is SYXK (Ji) 2011-006. All animals were housed under a 12-h light/dark cycle with free access to food and water in a colony room kept under a constant temperature (23 ± 1°C) and humidity (50 ± 5%). Mice in the chronic ethanol group were given intraperitoneal (i.p.) injection of ethanol (0.8 g/kg; 15% in saline), while the mice in the control group were given i.p. injection of the same volume of saline. Ethanol (95%) was diluted in saline to a final concentration of 15%. The i.p. injection was performed 1 time/day during 8:00–9:00 a.m. for 28 days. The electrophysiological recordings were performed 1 day after the last injection of ethanol.

### Anesthesia and Surgical Procedures

The anesthesia and surgical procedures have been described below (Chu et al., 2011a, b). The mice were anesthetized with urethane (1.3 g/kg body weight, i.p.) and were tracheotomized to avoid respiratory obstruction. On a custom-made stereotaxic frame, soft tissue was retracted to gain access to the dorsal portion of the occipital bone. A watertight chamber was created and a 1–1.5 mm craniotomy was drilled to expose the cerebellar surface corresponding to Crus II. The cerebellum surface was constantly superfused with oxygenated artificial cerebrospinal fluid (ACSF: 125 mM NaCl, 3 mM KCl, 1 mM MgSO_4_, 2 mM CaCl_2_, 1 mM NaH_2_PO_4_, 25 mM NaHCO_3_, and 10 mM D-glucose) with a peristaltic pump (Gilson Minipulse 3; Villiers-le-Bel, France) at 0.5 ml/min. Rectal temperature was monitored and maintained at 37.0 ± 0.2°C.

### Facial Stimulation and *in vivo* Electrophysiological Recording

Local field potential recordings from the GL were performed with an Axopatch-200B amplifier (Molecular Devices, Foster City, CA, United States). The potentials were acquired through a Digidata 1440 series analog-to-digital interface on a personal computer using Clampex 10.4 software (Molecular Devices). Recording pipettes were made with a puller (PB-10; Narishige, Tokyo, Japan) from a thick-walled borosilicate glass (GD-1.5; Narishige). Recording electrodes were filled with ACSF and with resistances of 3–5 MΩ. The recordings from the GL were performed at depths of 300–350 μm under the pia mater membrane.

Facial stimulation was performed by air-puff to the ipsilateral whisker pad through a 12-gauge stainless steel tube connected with a pressurized injection system (Picospritzer^®^ III; Parker Hannifin Co., Pine Brook, NJ, United States). The air-puff stimuli were controlled by a personal computer, were synchronized with the electrophysiological recordings, and were delivered at 0.05 Hz *via* a Master 8 controller (A.M.P.I., Jerusalem, Israel) and Clampex 10.4 software. For isolating mossy fiber-granule cell (MF-GC) synaptic transmission, picrotoxin (100 μM) was added to ACSF during all recordings to prevent GABA_A_ receptor-mediated inhibitory responses of Golgi cells. Single stimulation (60 ms, 60 psi) or stimuli train (20 Hz, 5-pulse, 10 ms, 60 psi) were used to evoke the MF-GC synaptic transmission in the absence of GABA_A_ receptors activity.

### Chemicals

Picrotoxin, (3,4-dihydro-2H-pyrano [2,3-b]quinolin-7-yl)-(cis-4-methoxy- cyclohexyl)-methanone [JNJ16259685 (JNJ)] and D-amino phosphono valeric acid (D-APV) were bought from Sigma-Aldrich (Shanghai, China). PEAQX and TCN 237 were purchased from Tocris (Bristol, United Kingdom). The drugs were dissolved in ACSF and applied directly onto the cerebellar surface by a peristaltic pump (0.5 ml/min).

### Data Analysis

The electrophysiological data were analyzed using Clampfit 10.4 software (Molecular Devices, Foster City, CA, United States). All data are expressed as the mean ± SEM. A one-way ANOVA (the Turkey *post hoc* test) and a two-way ANOVA (SPSS software) were used to determine the level of statistical significance among the groups of data. *P*-values below 0.05 were considered as statistically significant.

## Results

### Effect of Chronic Ethanol Exposure From Adolescence on the Facial Stimulation-Evoked Field Potential Response in Adult Mouse Cerebellar GL

**FIGURE 1:**
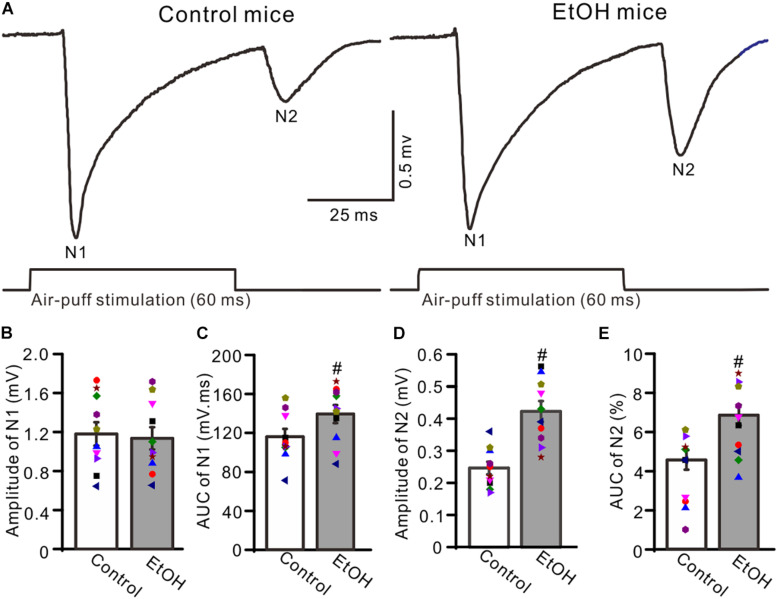
Chronic ethanol exposure enhanced the facial stimulation-evoked mossy fiber-granule cell (MF–GC) synaptic transmission in the mouse cerebellar cortex. **(A)** Representative field potential recording traces showing the facial stimulation (60 ms, 50 psi)-evoked responses in the granular layer (GL) of control and ethanol-exposed mice. **(B)** The mean and individual data (Symbols of different colors) show the amplitude of N1 in control and ethanol-exposed (EtOH) mice. **(C)** A bar graph with individual data showing the area under the curve (AUC) of N1 in control and ethanol-exposed (EtOH) mice. **(D,E)** Bar graphs and individual data showing the amplitude **(D)** and AUC **(E)** of N2 in control and ethanol-exposed (EtOH) mice. *n* = 10 in each group. #*P* < 0.05 vs. control.

To observe facial stimulation-evoked MF–GC synaptic transmission, we recorded the facial stimulation-evoked field potential response in the cerebellar GL in the presence of the GABA_A_ receptor antagonist picrotoxin (100 μM), which blocks the inhibitory components of Golgi cells. Blockade of GABA_A_ receptor activity and air-puff stimulation of the ipsilateral whisker pad (60 ms; 50–60 psi) evoked negative components N1 and N2 in the GL (Figure 1A), which were identified as components of facial stimulation-evoked MF–GC synaptic transmission (Bing et al., 2015a, b; Ma et al., 2019). To determine the effects of chronic ethanol exposure on facial stimulation-evoked MF–GC synaptic transmission, we compared the properties of the facial stimulation-evoked field potential response in the GL between chronic ethanol-exposed and non-ethanol-exposed (control) mice. Since there were no significant sex differences between the amplitudes of N1 and N2 in both control and ethanol-exposed mice, we pooled both the sexes for analysis. As shown in Figure 1, the N1 amplitude in the chronic ethanol-exposed group was 1.18 ± 0.12% mV (*n* = 10 mice), which was similar to that in the control group (1.13 ± 0.11% of the baseline value, *n* = 10 mice; *F* = 0.31, *P* = 0.67; Figures 1A,B). However, the area under the curve (AUC) of N1 was 138.2 ± 9.2% mV/ms (*n* = 10 mice) in the ethanol-exposed group, which was significantly larger than that in the control group (115.1 ± 7.9 mV/ms, *n* = 10 mice; *F* = 5.12, *P* = 0.016; Figure 1C). The N2 amplitude in the ethanol-exposed group was 0.42 ± 0.03 mV (*n* = 10 mice), which was significantly lower than that in the control group (0.25 ± 0.02 mV; *n* = 10 mice; *F* = 5.27, *P* = 0.003; Figure 1D). Moreover, the AUC of the N2 in the ethanol-exposed group was 6.85 ± 0.52 mV/ms (*n* = 10 mice), which was also significantly larger than that in the control group (4.56 ± 0.49 mV/ms; *n* = 10 mice; *F* = 4.41, *P* = 0.002; Figure 1E). These results indicate that the chronic ethanol exposure from adolescence induces a significant enhancement in the later components of facial stimulation-evoked MF–GC synaptic transmission in mice *in vivo*.

### Chronic Ethanol Exposure From Adolescent Enhanced the Facial Stimulation-Evoked MF-GC Synaptic Transmission via NMDARs

**FIGURE 2:**
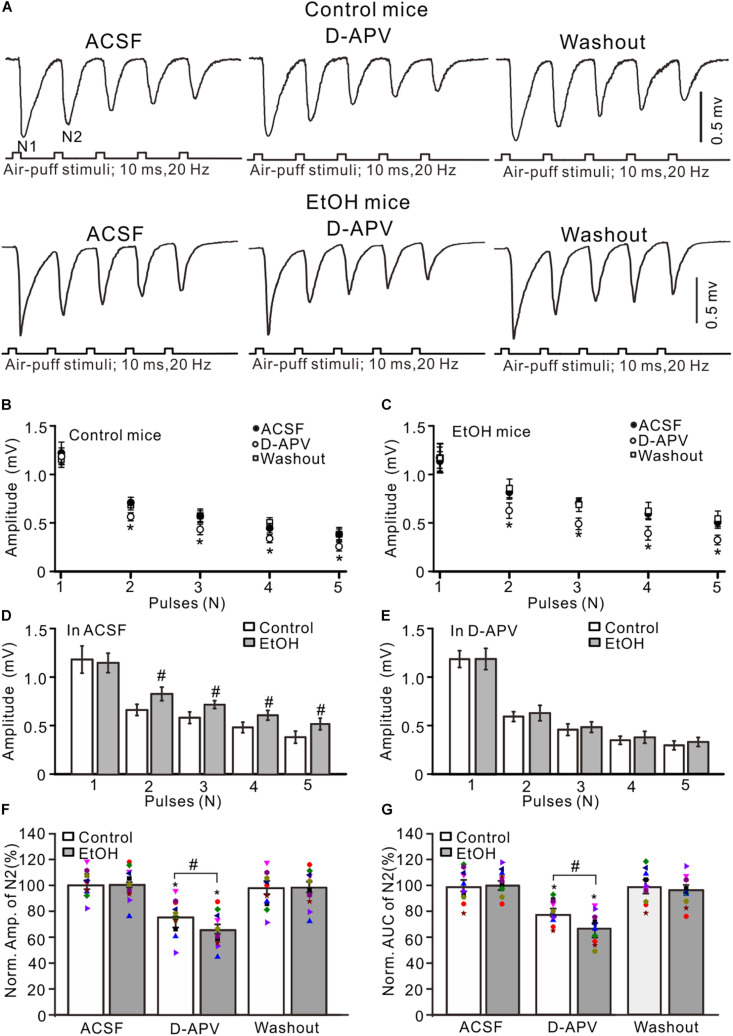
Blockade of N-methyl-D-aspartate receptors (NMDARs) prevented the chronic ethanol exposure-induced enhancement of facial stimulation-evoked MF–GC synaptic transmission. **(A)** Representative field potential traces showing that the air-puff stimuli (10 ms, 60 psi; 5 pulse, 20 Hz) on the ipsilateral whisker pad evoked field potential responses recorded from the GL of control **(upper)** and ethanol-exposed **(lower)** mice in treatments with artificial cerebrospinal fluid (ACSF), D-APV (250 μM), and recovery (washout). **(B)** Summary of data showing the absolute amplitudes of N1–N5 in treatments with ACSF, D-APV, and recovery (washout) in control mice. **(C)** Summary of data showing the absolute amplitudes of N1–N5 in treatments with ACSF, D-APV (250 μM), and recovery (washout) in ethanol-exposed mice. **(D)** A bar graph showing the mean amplitude of peaks recorded in the presence of ACSF in control and ethanol-exposed mice. **(E)** A bar graph showing the mean amplitude of peaks recorded in the presence of D-APV in control and ethanol-exposed mice. **(F)** A bar graph with individual data (Symbols of different colors) showing the normalized amplitude of N2 in control and ethanol-exposed (EtOH) mice in treatments with ACSF, D-APV, and recovery (washout). **(G)** A bar graphs with individual data (Symbols of different colors) showing the normalized AUC of N2 in control and ethanol-exposed (EtOH) mice in treatments with ACSF, D-APV, and recovery (washout). *n* = 10 in each group. *P < 0.05 vs. ACSF; #*P* < 0.05 vs. control.

Previous studies demonstrated that chronic ethanol exposure overdose impairs neuronal function *via* NMDARs (Nagy, 2004) and that NMDARs contribute to the later components of facial stimulation evoked by MF–GC synaptic transmission in the mouse cerebellar GL (Zhang et al., 2020). We examined the effect of adolescent chronic ethanol exposure on the NMDARs-mediated components of facial stimulation-evoked MF–GC synaptic transmission. To activate NMDARs in the cerebellar GL during facial stimulation-evoked MF–GC synaptic transmission, we employed a facial stimuli train (20 Hz, five pulses) to evoke five field potential responses (N1–N5) in the cerebellar GL (Zhang et al., 2020). Application of the selective NMDAR antagonist D-APV (250 μM) did not significantly affect the amplitude of N1 but induced a decrease in the amplitudes of N2–N5 in both the control (*P* < 0.05 vs. ACSF; Figures 2A,B) and the chronic ethanol-exposed mice (*P* < 0.05 vs. ACSF; Figures 2A,C). Indeed, the amplitudes of N2–N5 in the ethanol group were significantly higher than those in the control group in the presence of ACSF (*P* < 0.05; *n* = 10; Figure 2D), but they were not significantly different in the presence of D-APV (*P* > 0.05; *n* = 10; Figure 2E).

To understand the changes in the D-APV-sensitive components in the control and the ethanol-exposed mice, we compared the normalized value of N2. In the presence of D-APV, the normalized amplitude of N2 was 76.7 ± 4.2% of the baseline value (*F* = 5.26, *P* = 0.003 vs. ACSF; *n* = 10 mice; Figure 2F) in the control group and 64.9 ± 3.8% of the baseline value (*F* = 5.17, *P* = 0.0006 vs. ACSF; *n* = 10 mice; Figure 2F) in the ethanol-exposed group. The mean value of the normalized amplitude of N2 for ethanol-exposed mice was significantly lower than that in the presence of ACSF (*F* = 4.31, *P* = 0.032 vs. control; *n* = 10; two-way ANOVA; Figure 2F). Moreover, the normalized AUC of N2 was 68.5 ± 4.6% of the baseline value (*n* = 10 mice; *P* < 0.001; Figure 2G) in the control group and 55.3 ± 4.9% of the baseline value (*n* = 10 mice; *P* < 0.001; Figure 2G) in the ethanol-exposed group. The mean value of the normalized AUC of N2 in the ethanol-exposed group was significantly lower than that in the control group (*F* = 4.54, *P* = 0.012; *n* = 10 mice; two-way ANOVA; Figure 2G). These results indicate that the chronic ethanol exposure from adolescence augments the facial stimulation-evoked MF–GC synaptic transmission *via* NMDARs in adult mice *in vivo*.

### Chronic Ethanol Exposure From Adolescents Augmented the MF-GC Synaptic Transmission via GluN2A

**FIGURE 3:**
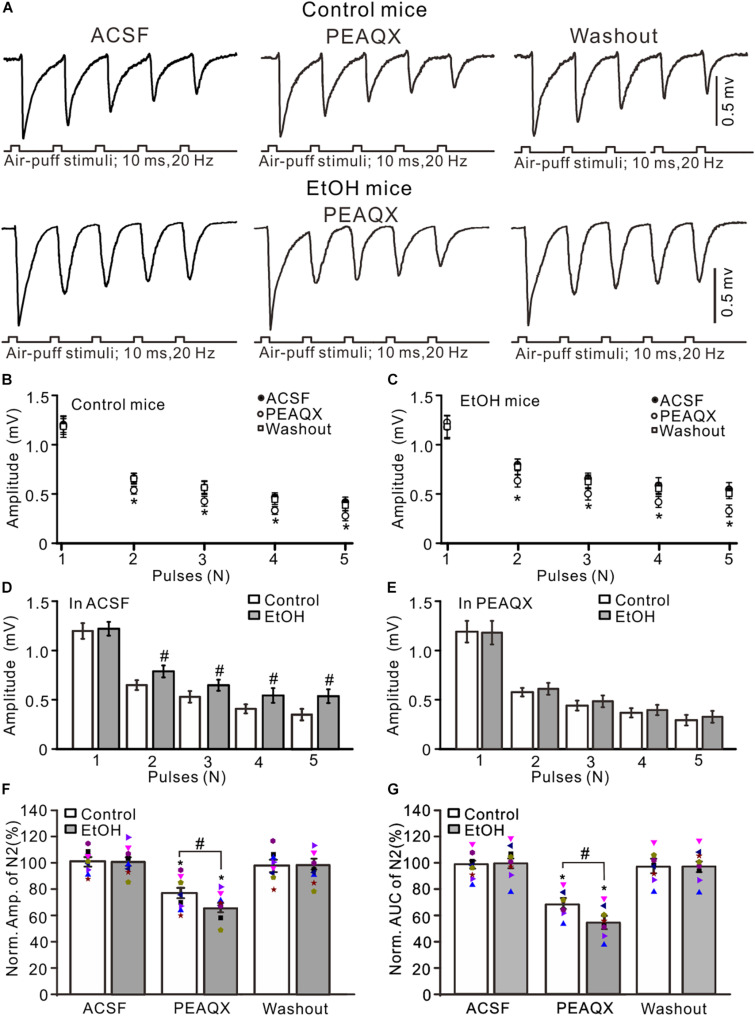
Blockade of GluN2A abolished the chronic ethanol exposure-induced enhancement of facial stimulation-evoked MF–GC synaptic transmission. **(A)** Representative field potential traces showing that the air-puff stimuli (10 ms, 60 psi; 5 pulse, 20 Hz) on the ipsilateral whisker pad evoked field potential responses, recorded from the GL of control **(upper)** and ethanol-exposed mice in treatments of ACSF, PEAQX (10 μM), and recovery (washout). **(B)** Summary of data showing the absolute (±SEM) amplitudes of N1–N5 in treatments with ACSF, PEAQX, and recovery (washout) in control mice. **(C)** Summary of data showing the absolute amplitudes of N1–N5 in treatments with ACSF, PEAQX, and recovery (washout) in ethanol-exposed mice. **(D)** Bar graph showing the mean amplitude of peaks recorded in the presence of ACSF in control and ethanol-exposed mice. **(E)** A bar graph showing the mean amplitude of peaks recorded in the presence of PEAQX in control and ethanol-exposed mice. **(F)** A bar graph with individual data (Symbols of different colors) showing the normalized amplitude of N2 in each treatment. **(G)** A bar graph with individual data (Symbols of different colors) showing the normalized AUC of N2 in treatments with ACSF, PEAQX, and recovery (washout). *n* = 8 in each group. *P < 0.05 vs. ACSF; #*P* < 0.05 vs. control.

GluN2A is expressed on the somas of GCs and the boutons of parallel fibers (Glitsch and Marty, 1999; Casado et al., 2000) and contributes to the facial stimulation-evoked MF–GC synaptic transmission in mice *in vivo* (Zhang et al., 2020). We observed the effect of a GluN2A blocker, PEAQX (10 μM), on the facial stimulation-evoked MF–GC synaptic transmission in the cerebellar GL of mice. Application of PEAQX did not significantly affect the amplitude of N1 but induced a significant decrease in the amplitude of N2–N5 in both the control (*P* < 0.05 vs. ACSF; Figures 3A,B) and ethanol-exposed mice (*P* < 0.05 vs. ACSF; Figures 3A,C). The amplitudes of N2–N5 in the ethanol-exposed group were significantly higher than those in the control group in the presence of ACSF (*P* < 0.05; *n* = 8 mice; Figure 3D) but were not significantly different in the presence of PEAQX (*P* > 0.05; *n* = 8; Figure 3E). In the presence of PEAQX, the normalized amplitude of N2 was 77.2 ± 3.9% of the baseline value (*F* = 7.23, *P* = 0.0004; *n* = 8 mice; Figure 3F) in the control group and 63.8 ± 3.5% of the baseline value (*F* = 6.85, *P* = 0.0007; *n* = 8 mice; Figure 3F) in the ethanol-exposed group. The mean normalized amplitude of N2 in the ethanol-exposed group was significantly higher than that in the control group (*F* = 5.18, *P* = 0.036; *n* = 8 mice two-way ANOVA; Figure 3F). Further, the normalized AUC of N2 was 68.6 ± 4.2% of the baseline value (*F* = 7.32, *P* = 0.0006; *n* = 8 mice; Figure 3G) in the control group and 56.5 ± 5.1% of the baseline value (*F* = 7.08, *P* = 0.0003; *n* = 8 mice; Figure 3G) in the ethanol-exposed group. The mean normalized AUC of N2 in the ethanol-exposed group was significantly larger than that in the control group (*F* = 5.17, *P* = 0.016; *n* = 8 mice; two-way ANOVA; Figure 3G).

**FIGURE 4:**
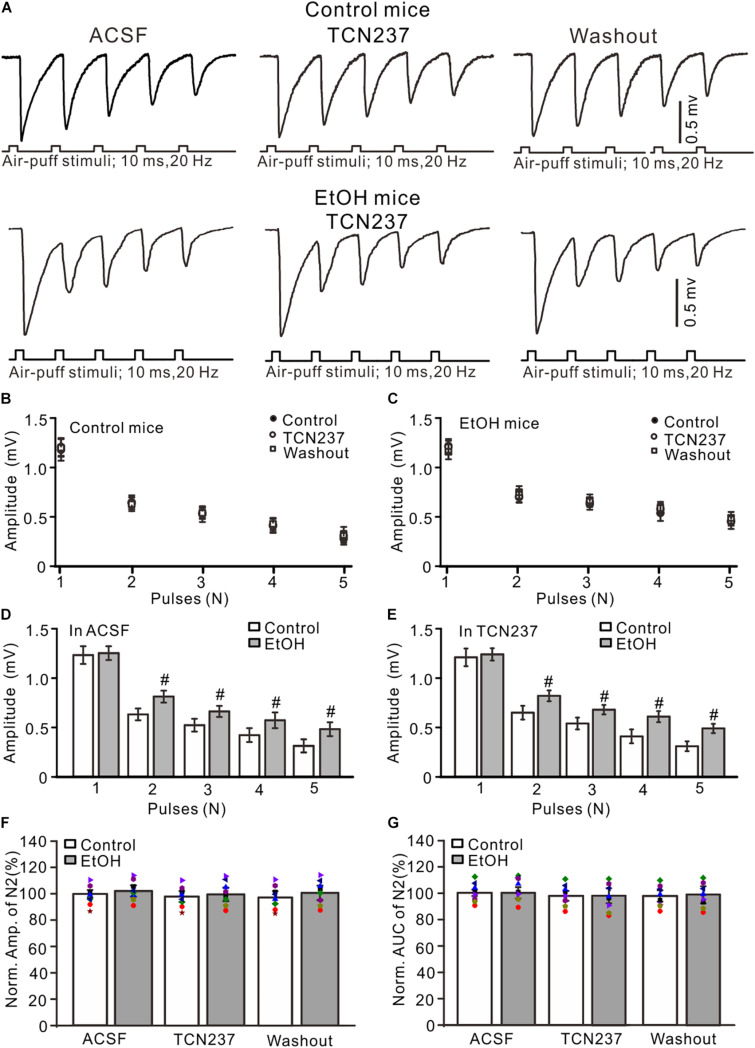
GluN2B blockade failed to prevent the ethanol exposure-induced enhancement of facial stimulation-evoked MF–GC synaptic transmission. **(A)** Representative field potential traces showing that the air-puff stimuli (10 ms, 60 psi; 5 pulse, 20 Hz) on the ipsilateral whisker pad evoked field potential responses, recorded from the GL of control **(upper)** and ethanol-exposed mice in treatments of ACSF, TCN (10 μM), and recovery (washout). **(B)** Summary of data showing the absolute amplitudes of N1–N5 in treatments with ACSF, TCN (10 μM), and recovery (washout) in control mice. **(C)** Summary of data showing the absolute amplitudes of N1–N5 in treatments with ACSF, TCN, and recovery (washout) in ethanol-exposed mice. **(D,E)** Bar graphs with individual data showing the normalized amplitude of N2 in ACSF **(D)** and in TCN237 mice. **(F,G)** Bar graphs with individual data (Symbols of different colors) showing the normalized amplitude **(F)** and AUC **(G)** of N2 in control and ethanol-exposed mice. *n* = 8 in each group. #*P* < 0.05 vs. control.

Since PEAQX is not sufficiently selective to distinguish between GluN2A and GluN2B (Frizelle et al., 2006), we used a selective GluN2B antagonist, TCN-237, to determine whether GluN2B contributed to facial stimulation-evoked MF–GC synaptic transmission in the cerebellar GL (McCauley et al., 2004). Perfusion of TCN-237 (10 μM) did not significantly change the amplitude of N1–N5 in either the control (*P* > 0.05 vs. ACSF; Figures 4A,B) or the ethanol-exposed mice (*P* > 0.05 vs. ACSF; *n* = 8; Figures 4A,C). However, the amplitudes of N2–N5 in the ethanol-exposed group were significantly higher than those in the control group in the presence of ACSF (*P* < 0.05; *n* = 8; Figure 4D) as well in the presence of TCN-237 (*P* < 0.05; *n* = 8; Figure 4E). In the presence of TCN-237, the normalized amplitude of N2 was 98.3 ± 4.1% of the baseline value (*F* = 0.19, *P* = 0.72 vs. ACSF; *n* = 8 mice; Figure 4F) in the control group and 97.4 ± 5.1% of the baseline value (*F* = 0.22, *P* = 0.65; *n* = 8 mice; Figure 4F) in the ethanol-exposed group. The mean normalized amplitude of N2 in the ethanol-exposed group was similar to that in the control group (*F* = 0.18, *P* = 0.67; *n* = 8 mice two-way ANOVA; Figure 4F). Moreover, blockade of GluN2B did not significantly change the AUC of N2 in either the control or the ethanol-exposed mice. The normalized AUC of N2 was 97.7 ± 3.6% of the baseline value (*F* = 0.18, *P* = 0. 67 vs. ACSF; *n* = 8 mice; Figure 4C) in the control group and 98.1 ± 5.7% of the baseline value (*F* = 0.16, *P* = 0.71 vs. ACSF; *n* = 8 mice; Figure 4C) in the ethanol-exposed group. The normalized AUC of N2 in the ethanol-exposed group was not significantly different from that in the control group (*F* = 0.21, *P* = 0.62; *n* = 8 mice two-way ANOVA; Figure 4G). These results indicate that chronic ethanol exposure from adolescence augments the NMDAR-sensitive components during the facial stimulation-evoked MF–GC synaptic transmission but not *via* GluN2B.

### Blocking mGluR1 Failed to Prevent Chronic Ethanol-Induced Enhancement of Facial Stimulation-Evoked MF–GC Synaptic Transmission

**FIGURE 5:**
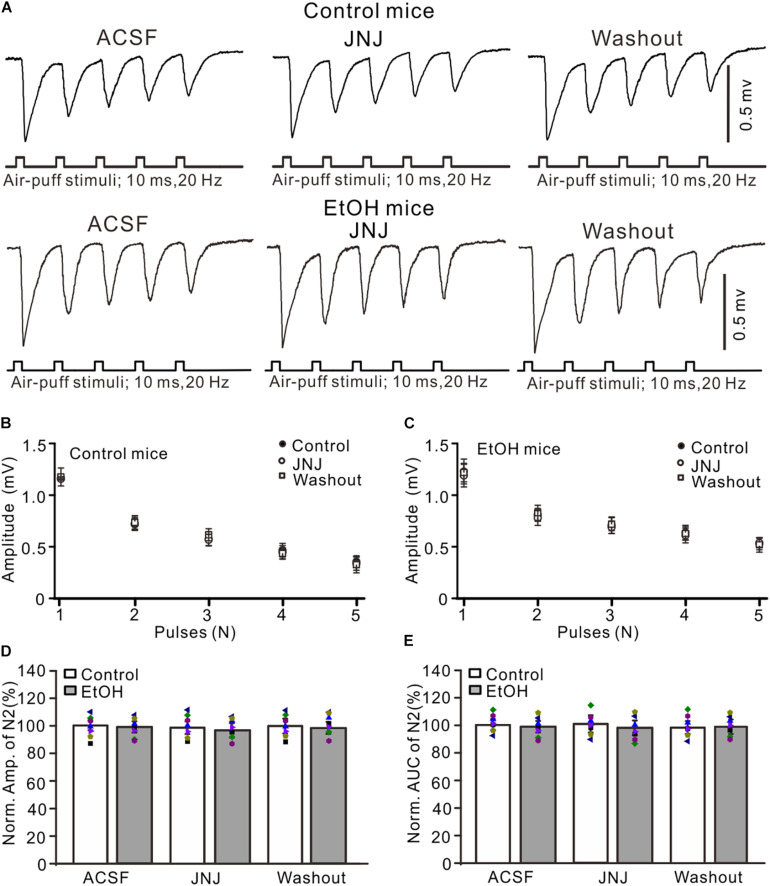
Blockade of mGluR1 failed to prevent the ethanol exposure-induced enhancement of facial stimulation-evoked MF–GC synaptic transmission. **(A)** Representative field potential traces showing that the air-puff stimuli (10 ms, 60 psi; 5 pulse, 20 Hz) on the ipsilateral whisker pad evoked field potential responses, recorded from the GL of control and ethanol-exposed mice in treatments of ACSF, JNJ (10 μM), and recovery (washout). **(B)** Summary of data showing the absolute amplitudes of N1–N5 in treatments with ACSF, JNJ, and recovery (washout) in control mice. **(C)** Pooled data showing the absolute values of N1–N5 in treatments with ACSF, JNJ, and recovery (washout) in ethanol-exposed mice. **(D)** A bar graph with individual data (Symbols of different colors) showing the normalized amplitude of N2 in treatments with ACSF, JNJ, and recovery (washout). **(E)** A bar graph with individual data (Symbols of different colors) showing the normalized AUC of N2 for each treatment. *n* = 7 in each group.

Chronic ethanol exposure could enhance the expression and function of metabolic glutamate receptor 1 (mGluR1) in several regions of the brain (Cozzoli et al., 2014), and activation of mGluR1 may contribute to the chronic ethanol-induced enhancement of facial stimulation-evoked MF–GC synaptic transmission. Therefore, we examined whether mGluR1 contributed to the chronic ethanol exposure-induced enhancement of facial stimulation-evoked MF–GC synaptic transmission. Administration of a selective mGluR1 receptor antagonist, JNJ16259685 (10 μM), did not induce a significant change in the amplitude of N1–N5 in the control (Figures 5A,B) and the ethanol-exposed mice (Figures 5A,C). In the presence of JNJ16259685, the normalized amplitude of N2 was 99.3 ± 5.3% of the baseline value (*F* = 0.16, *P* = 0.65; *n* = 7 mice; Figure 5D) in the control group and 98.4 ± 4.6% of the baseline value (*F* = 0.23, *P* = 0.78; *n* = 7 mice; Figure 5D) in the ethanol-exposed group. The mean normalized amplitude of N2 in the ethanol-exposed group was not significantly different from that in the control group (*F* = 0.26, *P* = 0.63; two-way ANOVA; *n* = 7 mice; Figure 5D). Moreover, the normalized AUC of N2 was 99.6 ± 5.8% of the baseline value (*F* = 0.12, *P* = 0.76; *n* = 7 mice; Figure 5E) in the control group and 97.7 ± 5.3% of the baseline value (*F* = 0.13, *P* = 0.73; *n* = 7 mice; Figure 5E) in the ethanol-exposed group. These results indicate that blockade of mGluR1 does not prevent the chronic ethanol exposure-induced enhancement of facial stimulation-evoked MF–GC synaptic transmission in mice *in vivo*.

## Discussion

In this study, we found that the chronic ethanol exposure from adolescence led to an enhancement in the facial stimulation-evoked MF–GC synaptic transmission, which was blocked by a selective NMDAR antagonist. The chronic ethanol exposure-induced enhancement of the facial stimulation-evoked MF–GC synaptic transmission was prevented by a selective GluN2A blocker but was not prevented by a selective GluN2B antagonist or a selective mGluR1 blocker. These results indicate that the chronic ethanol exposure from adolescence enhances facial stimulation-evoked MF–GC synaptic transmission *via* GluN2A, which suggests that chronic ethanol exposure from adolescence may impair high-fidelity properties during the sensory information processing in the cerebellar cortical GL.

Chronic ethanol exposure can affect neurotransmitter release, synaptic transmission, and neural circuit plasticity in multiple brain regions. The latter is related to tolerance and dependence in human (Tsai and Coyle, 1998; Koob, 2003; Heilig et al., 2010; Koob and Volkow, 2010, 2016; Wu et al., 2014; Li et al., 2019). Heavy chronic ethanol consumption from adolescence to adulthood was shown to significantly impair the motor performance in female rats, inducing spontaneous locomotor activity deficits, bradykinesia, incoordination, motor learning disruption, and atrophy and neuronal loss in the cerebellum (Maldonado-Devincci et al., 2021). Behavioral studies have shown that adolescents and adults have different behavioral sensitivities to alcohol and that the cerebellum of adolescents is more sensitive to ethanol exposure (Best and Miller, 2010; Spear, 2016). Chronic ethanol exposure in adolescence could impair the expression of long-term synaptic potentiation in animals (Goodwani et al., 2017). The core of understanding the pathophysiology of ethanol dependence is describing the specific neural adaptations in glutamatergic and GABAergic synaptic transmissions caused by chronic ethanol exposure. The effects of chronic ethanol exposure on glutamatergic signal transduction are mainly concentrated on postsynaptic glutamate receptors (David and Marisa, 2013; Bliss et al., 2014), which affect ionic receptors and various metabotropic glutamate receptor subtypes.

In the cerebellar cortex, GCs receive and respond to sensory information conveyed by MFs (Huang et al., 2013; Ishikawa et al., 2015) and Golgi cells set the spiking threshold and therefore the number of different afferents required to drive GC firing by offering feed-forward inhibition (Marr, 1969; D’Angelo et al., 2013). To isolate the effect of chronic ethanol exposure on MF–GC excitatory synaptic transmission, we applied a GABAA receptor antagonist to the cerebellar surface to block Golgi cell–GC GABAergic transmission. The results showed that chronic ethanol exposure significantly enhanced facial stimulation-evoked MF–GC synaptic transmission in the absence of the GABAA receptor activity, which suggests that the chronic ethanol exposure modulates the MF–GC excitatory synaptic transmission in mice *in vivo*.

N-methyl-D-aspartate receptors are a crucial target of chronic ethanol exposure in the central nervous system and are involved in tolerance, dependence, withdrawal, craving, and relapse (Woodward, 2000; Pignataro et al., 2009). Acute ethanol exposure leads to inhibitory actions on the activity of NMDARs, which has been illustrated previously in the slices of several brain regions (Calton et al., 1999; Ronald et al., 2001; Yaka et al., 2003; Boikov et al., 2020). Chronic ethanol exposure in adult animals induces upregulation of the number and function of NMDARs and also increases the expression of NMDAR subunits (Chandler et al., 1993; Follesa and Ticku, 1995; Hoffman et al., 1996). Many studies found that chronic ethanol exposure could significantly enhance the function of NMDARs and its mediated glutamatergic synaptic transmission (Gulya et al., 1991; Smothers et al., 1997; Grover et al., 1998; Cebere et al., 1999; Lack et al., 2007) and that chronic ethanol exposure activates NMDARs to a greater extent than the other ionic glutamatergic receptors (Gulya et al., 1991; Chandler et al., 1997, 1999; Smothers et al., 1997). Chronic ethanol exposure was shown to produce a long-term increase in the activity of NR2B-containing NMDARs in the dorsomedial striatum of rats (Wang et al., 2007, 2010). Consistent with the findings of previous studies (Gulya et al., 1991; Smothers et al., 1997; Grover et al., 1998; Cebere et al., 1999; Lack et al., 2007; Wang et al., 2007, 2010), the results of the present study showed that chronic ethanol exposure induced augmentation of facial stimulation-evoked MF–GC synaptic transmission was prevented by an NMDAR antagonist, which suggests that chronic ethanol exposure enhances the activity of NMDARs. In addition, the chronic ethanol exposure-induced facilitation of N2–N5 responses might be attributed to an increased probability of glutamate release from the presynaptic terminals during the facial stimulation-evoked MF–GC synaptic transmission.

In a previous study, NR2A and NR2C mRNA were detected in cerebellar GCs during the second postnatal week, whereas the NR2B mRNA was transiently expressed in the GCs during the first 2 postnatal weeks in rats (Akazawa et al., 1994). GluN2A has been detected on the somas of the GCs and boutons of the parallel fibers (Glitsch and Marty, 1999; Casado et al., 2000) and contributes to facial stimulation-evoked MF–GC synaptic transmission in mice *in vivo* (Zhang et al., 2020). It has been demonstrated that the GluN2C subunit was preferentially incorporated into triheteromeric GluN1/GluN2A/GluN2C receptors in cerebellar GCs, which might contribute to MF–GC synaptic transmission (Bhattacharya et al., 2018). However, our results showed that blockade of GluN2A induced a strong depression of MF–GC synaptic transmission in chronic ethanol-exposed mice; however, the enhancement of MF–GC synaptic transmission in chronic ethanol-exposed mice was not affected by the blockade of GluN2B. This result indicates that chronic ethanol exposure augments the NMDAR-sensitive components of facial stimulation-evoked MF–GC synaptic transmission *via* GluN2A but not *via* GluN2B. Previous studies also showed that the NR2B mRNA expression was significantly increased after chronic ethanol exposure (Follesa and Ticku, 1995; Hu et al., 1996; Snell et al., 1996; Roberto et al., 2006; Kash et al., 2009), particularly in the prefrontal cortex and in the hippocampus of alcoholics (Zhou et al., 2011; Farris and Mayfield, 2014). NR2B or/and NR2A protein expression was found to be increased in rodent brains after chronic ethanol exposure (Snell et al., 1996; Läck et al., 2005; Kash et al., 2008, 2009; Obara et al., 2009). Ethanol exposure led to an increase in the activity of Fyn kinase in the dorsomedial striatum of rats, resulting in an enhancement of NR2B phosphorylation and long-term facilitation of its activity (Wang et al., 2007, 2010). In addition, chronic ethanol exposure has been found to facilitate mGluR1 function in the cerebellum (Cozzoli et al., 2014). However, our results showed that the blockade of mGluR1 failed to prevent the chronic ethanol exposure-induced enhancement of facial stimulation-evoked MF–GC synaptic transmission in mice *in vivo*.

Collectively, we can conclude that chronic ethanol exposure in adolescence enhances facial stimulation-evoked MF–GC synaptic transmission *via* GluN2A, which might impair the high-fidelity properties of the sensory information transfer in the cerebellar GL in adult mice. Our results provide evidence for the further understanding of cellular and synaptic mechanisms of chronic ethanol exposure and their effects on motor coordination, motor learning, and cognitive functions from adolescence.

## Data Availability Statement

The original contributions presented in the study are included in the article/supplementary material, further inquiries can be directed to the corresponding author/s.

## Ethics Statement

The animal study was reviewed and approved by the Animal Care and Use Committee of the Yanbian University.

## Author Contributions

D-LQ, B-XL, G-HD, and S-BC conceived and designed the experiments. B-XL, G-HD, H-LL, J-SZ, and Y-HB performed the experiments. C-PC and D-LQ analyzed the data. Y-HB contributed to reagents, materials, and analysis tools. D-LQ, C-PC, and S-BC wrote the manuscript. All authors contributed to the article and approved the submitted version.

## Conflict of Interest

The authors declare that the research was conducted in the absence of any commercial or financial relationships that could be construed as a potential conflict of interest.

## Publisher’s Note

All claims expressed in this article are solely those of the authors and do not necessarily represent those of their affiliated organizations, or those of the publisher, the editors and the reviewers. Any product that may be evaluated in this article, or claim that may be made by its manufacturer, is not guaranteed or endorsed by the publisher.
